# Cerebrospinal Fluid CXCL10 as a Candidate Surrogate Marker for HTLV-1-Associated Myelopathy/Tropical Spastic Paraparesis

**DOI:** 10.3389/fmicb.2019.02110

**Published:** 2019-09-11

**Authors:** Keiko Tamaki, Tomoo Sato, Jun Tsugawa, Shinsuke Fujioka, Naoko Yagishita, Natsumi Araya, Junji Yamauchi, Ariella L. G. Coler-Reilly, Misako Nagasaka, Yasuhiro Hasegawa, Yoshihisa Yamano, Yoshio Tsuboi

**Affiliations:** ^1^Department of Neurology, Fukuoka University, Fukuoka, Japan; ^2^Department of Rare Diseases Research, Institute of Medical Science, St. Marianna University School of Medicine, Kawasaki, Japan; ^3^Department of Oncology, Karmanos Cancer Institute, Wayne State University, Detroit, MI, United States; ^4^Department of Advanced Medical Innovation, St. Marianna University Graduate School of Medicine, Kawasaki, Japan; ^5^Department of Neurology, St. Marianna University School of Medicine, Kawasaki, Japan

**Keywords:** HTLV-1, HAM/TSP, cerebrospinal fluid, neopterin, CXCL10, biomarker

## Abstract

Human T-cell leukemia virus type 1 (HTLV-1)-associated myelopathy/tropical spastic paraparesis (HAM/TSP) is a debilitating, progressive disease without effective treatment; therefore, development of disease modifying therapy that improves long-term functional outcomes is an unmet need for patients. However, it is virtually impossible to consider this as a primary endpoint in clinical trials owing to the prolonged disease course. Therefore, development of surrogate markers that help predict the effectiveness of new interventions is essential. Currently, several candidate surrogate markers have been identified for HAM/TSP. Cerebrospinal fluid (CSF) C-X-C motif chemokine 10 (CXCL10) is involved in the pathogenesis of HAM/TSP and was shown to correlate with disease progression. However, it remains unclear whether changes in CSF CXCL10 levels are observed in response to treatment and whether these correlate with prognosis. Here we investigated several markers, including CSF CXCL10, in this respect. Data pertaining to patient characteristics and results of motor function evaluation and CSF examination of 13 HAM/TSP patients who received steroid treatment were retrospectively analyzed. Osame motor disability scores (OMDS), 10 m walking time, and CSF levels of CXCL10, neopterin, total protein, cell counts, and anti-HTLV-1 antibody titer were compared before and after steroid therapy. Levels of all CSF markers, with the exception of cell count, were significantly decreased after treatment. Nine of the 13 patients (69.2%) showed improvement in OMDS and were considered responders. Pre-treatment CSF levels of CXCL10 and anti-HTLV-1 antibody titer in responders were higher than those in non-responders (*p* = 0.020 and *p* = 0.045, respectively). Patients who continued low-dose oral prednisolone maintenance therapy after methylprednisolone pulse therapy showed sustained improvement in OMDS and CSF CXCL10 and neopterin levels lasting for 2 years. In contrast, OMDS and the CSF marker levels in patients who discontinued treatment returned to pre-treatment levels. This rebound phenomenon was also observed in patients who discontinued oral prednisolone therapy independently of pulse therapy. Our findings suggest that CSF CXCL10 may serve as a therapy-response and therapy-predictive marker for HAM/TSP. In addition, since decrease in CSF CXCL10 level was associated with good functional prognosis, CSF CXCL10 is a potential surrogate marker for treatment of HAM/TSP.

## Introduction

Human T-cell leukemia virus type 1 (HTLV-1) is a human pathogenic retrovirus (Poiesz et al., 1980). A proportion of HTLV-1 carriers develop adult T-cell leukemia/lymphoma (Uchiyama et al., 1977; Hinuma et al., 1981) and/or HTLV-1-associated myelopathy/tropical spastic paraparesis (HAM/TSP) (Gessain et al., 1985; Osame et al., 1986). HAM/TSP is a neuroinflammatory disease characterized by infiltration of HTLV-1-infected T-cells into the spinal cord; the resultant chronic inflammation is believed to lead to spinal cord damage (Yamano and Sato, 2012; Bangham et al., 2015). The disease is characterized by spastic paraparesis, bladder and rectal disturbance, and sensory abnormality. The symptoms typically last throughout the life of the afflicted individual. Therefore, the true endpoint of treatment for HAM/TSP is the improvement of long-term functional prognosis. Gait disturbance, which is the main symptom of HAM/TSP, generally worsens over several years (Olindo et al., 2006; Martin et al., 2010; Coler-Reilly et al., 2016); therefore, a clinical trial with substantially long follow- up is required to prove the efficacy of treatment for the true endpoint. However, such trials are difficult to conduct and cannot immediately deliver new drugs to patients suffering from illness. Therefore, instead of this true endpoint, it is necessary to identify a surrogate endpoint that can facilitate quicker evaluation of the therapeutic efficacy in patients with HAM/TSP. This is a significant unmet need for patients with HAM/TSP.

An ideal surrogate marker should qualify the following criteria (Buyse et al., 2010; Zhao et al., 2015):

1. good correlation with disease progression;
2. biological plausibility that shows association with the true endpoint;
3. correlation with the true endpoint independent of treatment; and
4. the effect of treatment on the surrogate marker correlates with its effect on the true endpoint.

Several candidate surrogate markers in the context of HAM/TSP have already been identified; these include Cerebrospinal fluid (CSF) markers (neopterin, C-X-C motif chemokine 10 (CXCL10), anti-HTLV-1 antibody titer, cell count, and total protein) and HTLV-1 proviral load (Matsuzaki et al., 2001; Olindo et al., 2005; Sato et al., 2013; Matsuura et al., 2016). Among these, CXCL10 (a chemokine) and neopterin (a metabolite of guanosine triphosphate) are abundantly present in the CSF of HAM/TSP patients, and their concentrations were shown to correlate with the degree of disease progression (Nomoto et al., 1991; Narikawa et al., 2005; Sato et al., 2013). In particular, CXCL10 has been implicated in the causative mechanism of chronic inflammation in HAM/TSP (Ando et al., 2013; Araya et al., 2014). CXCL10 produced by astrocytes in the spinal cord of HAM/TSP patients recruits CXCR3-positive cells (including HTLV-1-infected cells and inflammatory cells) into the spinal cord; in addition, interferon γ produced by the recruited cells further induces CXCL10 production from astrocytes in the spinal cord lesions (Ando et al., 2013). Therefore, CSF CXCL10 is one of the key players in the pathogenesis of HAM/TSP. Furthermore, patients with high levels of CSF CXCL10 were shown to have greater disease activity and poorer long-term prognosis as compared with their counterparts with low CSF CXCL10 levels (Sato et al., 2018b). Thus, CSF CXCL10 qualifies criteria 1–3 for surrogate markers. However, whether it qualifies criterion 4 is not clear. In other words, whether decrease in CSF CXCL10 concentration induced by treatment is associated with improved long-term functional prognosis is not clear. Therefore, in the present study, we first examined whether candidate markers including CSF CXCL10 change in response to treatment. Second, we aimed to verify whether the change in marker level is related to the clinical course for at least 2 years.

Corticosteroids are commonly used to treat HAM/TSP in endemic areas (Bangham et al., 2015). These are effective in suppressing inflammation, especially in the spinal cord. There are two main steroid therapies for patients with HAM/TSP: oral prednisolone therapy and intravenous high-dose methylprednisolone pulse therapy. The effectiveness of oral prednisolone therapy was shown in a retrospective study that included a control group (Coler-Reilly et al., 2017). However, there is no clear consensus on the effectiveness of methylprednisolone pulse therapy (Duncan and Rudge, 1990; Araujo et al., 1993; Nakagawa et al., 1996). Even in studies that demonstrated its effectiveness, the beneficial effects were only transient (Duncan and Rudge, 1990; Croda et al., 2008). There have been no reports about the need for maintenance therapy so far. In addition, it is not clear as to which category of patient is suitable for this steroid treatment. We hypothesized that pulse therapy is more effective for patients with high disease activity, and oral prednisolone treatment is necessary as an additional treatment in order to sustain its effect. In this study, we sought to examine the effectiveness of these steroid therapies and to identify markers that predict therapeutic response; in addition, we sought to clarify the group of patients suitable for this treatment.

In this study, we retrospectively investigated the clinical course and the time course of CSF marker levels (including CXCL10) in 22 HAM/TSP patients (13 patients who received methylprednisolone pulse therapy, five patients who did not receive any steroid therapy, and four patients who discontinued low-dose oral prednisolone therapy). Next, we sought to identify patients who are more likely to benefit from steroid therapy; for this purpose, we compared the pre-treatment marker levels between patients who showed improvement in Osame motor disability scores (OMDS) post-treatment and those who did not show improvement post-treatment. Furthermore, in order to examine the linkage between the clinical course and the chronological changes in CSF marker levels, we investigated three groups: patients who received prednisolone maintenance therapy after methylprednisolone pulse therapy, patients who did not receive prednisolone maintenance therapy, and independently of pulse therapy, patients who discontinued oral prednisolone therapy.

## Materials and Methods

### Ethical Considerations

The study was approved by the Institutional Review Board of St. Marianna University School of Medicine (#1646) and Fukuoka University Hospital (#14-2-08). Prior to the collection of blood or CSF samples, all participants provided written informed consent for analysis of their samples for research purposes as part of their clinical care.

### Study Design and Subjects

This was a retrospective observational study. All subjects were diagnosed with HAM/TSP based on the World Health Organization criteria (Osame, 1990). The clinical characteristics and treatment details of all subjects are summarized in Table 1. The treatment group composed of 13 HAM/TSP patients; this included seven patients who received methylprednisolone pulse therapy at the Fukuoka University Hospital between April 2012 and August 2014 (patient nos. 1–7) and six patients who received methylprednisolone pulse therapy at the St. Marianna University Hospital between February 2012 and May 2014 (patient nos. 8–13). The untreated group included five HAM/TSP patients who underwent CSF examination at two time points and who did not receive steroids or interferon-α between the two time points (patient nos. 14–18). As a separate analysis group, we used the data from 4 HAM/TSP patients who had received and subsequently discontinued oral prednisolone therapy (patient nos. 19–22).

**TABLE 1:** Characteristics of patients with HAM/TSP enrolled in this study.

**Hospital name**	**Patient no.**	**Age (years)**	**Sex**	**Disease duration**	**OMDS at baseline**	**mPSL dose (mg/day)**	**PSL dose from after pulse therapy until year 2 (mg/day)**
Fukuoka University Hospital (FUH)	1	61	F	3 months	6	1000	0.0
	2	71	F	8 months	9	1000	50.0 → 10.0
	3	71	F	6 months	4	1000	0.0
	4	72	F	2 years	5	1000	0.0^∗^
	5	48	F	16 years	4	1000	0.0
	6	67	F	4 years	4	1000	20.0 → 5.0
	7	68	F	9 years	4	1000	0.0
St. Marianna University Hospital (SMUH)	8	58	F	1 year	6	1000	20.0 → 10.0
	9	53	M	2 years	6	250	40.0 → 7.0
	10	67	F	4 years	3	500	15.0 → 2.0
	11	33	F	17 years	6	500	20.0 → 5.0
	12	67	F	19 years	8	500	30.0 → 8.0
	13	62	F	10 years	6	1000	25.0 → 7.0
SMUH	14	71	F	18 years	5	0	N/A
	15	71	F	13 years	5	0	N/A
	16	59	F	1 year	1	0	N/A
	17	64	F	4 years	4	0	N/A
	18	45	F	10 years	4	0	N/A

**Hospital name**	**Patient no.**	**Age (years)**	**Sex**	**Disease duration**	**OMDS at baseline**	**PSL dose (mg/day)**	

FUH	19	58	F	3 years	3	5	
SMUH	20	59	F	12 years	5	3	
	21	64	F	18 years	5	3	
	22	67	F	4 years	4	5	

*OMDS, osame motor disability score; mPSL, methylprednisolone; PSL, prednisolone; F, female; M, male; N/A, not applicable. ^∗^Patient no. 4 received oral prednisolone therapy from 3 months after treatment because of high levels of CXCL10 and neopterin (Figure 3).*

### Treatment Details

At the Fukuoka University Hospital, methylprednisolone 1000 mg per day was instilled intravenously for three consecutive days; after a gap of 4 days, methylprednisolone 1000 mg per day was infused intravenously again for three consecutive days. Directly after the pulse therapy, two patients received oral prednisolone therapy whereas five patients did not receive oral prednisolone therapy (Table 1). At the St. Marianna University Hospital, methylprednisolone (250, 500, or 1000 mg per day) was instilled intravenously for three consecutive days; subsequently, all patients (*n* = 6) received oral prednisolone therapy (Table 1). Since the dose of oral prednisolone was gradually tapered, Table 1 shows both the starting dose and the 2-year dose. In this paper, a series of treatments implemented at the two hospitals are collectively described as “steroid therapy.” In four patients (nos. 19–22), 3–5 mg of oral prednisolone was administered daily for at least 6 months.

### Disease Evaluation

Data pertaining to OMDS, (Table 2) and 10 m timed walk were collected as clinical outcome measures. The OMDS was evaluated before treatment and 1 month after treatment at both the university hospitals. Subjects whose OMDS improved 1 month after treatment compared with that at baseline were defined as responders, and those who did not show improvement were defined as non-responders. Subsequently, OMDS was measured every month for at least 6 months. Only patients who were able to walk for 10 m with or without walking aids underwent the 10 m timed walk. We could collect the data on 10 m timed walk before and about 2 weeks after treatment was performed in both hospitals. Since the 10 m timed walk was not performed regularly at the Fukuoka University Hospital, there are many missing data in this respect.

**TABLE 2:** Osame motor disability score.

**Grade**	**Motor disability**
0	No walking or running abnormalities
1	Normal gait but runs slowly
2	Abnormal gait (stumbling, stiffness)
3	Unable to run
4	Needs handrail to climb stairs
5	Needs a cane (unilateral support) to walk
6	Needs bilateral support to walk
7	Can walk 5–10 m with bilateral support
8	Can walk 1–5 m with bilateral support
9	Cannot walk, but able to crawl
10	Cannot crawl, but able to move using arms
11	Cannot move around, but able to turn over in bed
12	Cannot turn over in bed
13	Cannot even move toes

### Measurement of Biomarkers

Data pertaining to the following biomarkers were collected: CSF markers (CXCL10, neopterin, total protein, anti-HTLV-1 antibody titer, and cell counts) and HTLV-1-proviral load in peripheral blood mononuclear cells (PBMCs). In both hospitals, lumbar puncture for CSF examination is performed before and about 2 weeks after the start of treatment (mean ± standard deviation (SD): 2.5 ± 0.9 weeks from the first day of pulse therapy). CSF samples and PBMCs were prepared as described previously (Sato et al., 2013). Briefly, CSF obtained by lumbar puncture was used for routine laboratory tests and further analysis. The anti-HTLV-1 antibody titer in CSF was determined using the gelatin particle agglutination test (Serodia-HTLV-1; Fujirebio, Tokyo, Japan). Neopterin level in CSF was measured using high-performance liquid chromatography at a commercial laboratory (SRL Inc., Tokyo, Japan). CXCL10 in CSF was measured using a cytometric bead array (BD Biosciences, Franklin Lakes, NJ, United States). PBMCs were isolated with standard procedures using Pancoll density gradient centrifugation (density 1.077 *g*/mL; PAN-Biotech GmbH, Aidenbach, Germany). HTLV-1 proviral load was measured using real-time PCR, following DNA extraction from PBMCs, as previously described (Yamano et al., 2002).

### Statistical Analysis

The Wilcoxon signed rank test was used to compare the pre- and post-treatment biomarker levels in the same patient. The Mann–Whitney U test was used to compare baseline values of biomarkers between two patient groups (responders and non-responders). Fisher’s exact test was used to examine the relationship between additional oral prednisolone therapy and the change in OMDS during the observation period. Statistical analyses and graph composition were performed using R version 3.2.2 (R Foundation for Statistical Computing, Vienna, Austria) or GraphPad Prism 7 (GraphPad Software, Inc., San Diego, CA, United States). All *p*-values are two tailed, and the threshold of significance was set at 0.05.

## Results

### Subject Characteristics

The median age (range) of 13 HAM/TSP patients (patient nos. 1–13; 1 male and 12 females) who received methylprednisolone pulse therapy in the treatment group was 67 (33–72) years; the median (range) baseline OMDS was 6 (3–9) (Table 1). We defined rapid progressors as those who developed OMDS grade 4 or above within 1 year or developed OMDS grade 5 or above within 2 years from the onset of motor symptoms. The percentage of rapid progressors was 46.2% (6 out of 13 patients); these included four of seven patients at the Fukuoka University Hospital (patient nos. 1–4) and two of six patients at the St. Marianna University Hospital (patient nos. 8 and 9). The characteristics of five HAM/TSP patients (patient nos. 14–18; all females) in the untreated group were median age (range), 64 (45–71) years; median (range) baseline OMDS, 4 (1–5). There were no rapid progressors in this group. In a separate analysis group (4 HAM/TSP patients; nos. 19–22; all females) who received low-dose oral prednisolone therapy, the median age (range) was 61.5 (58–67) years and the median (range) baseline OMDS was 4.5 (3–5). There were also no rapid progressors in this group.

### Effect of Steroid Therapy on Lower Limb Motor Function

The percentage of patients whose OMDS improved by ≥1 after 1 month from the start of steroid therapy was 57.1% (four of seven patients) at the Fukuoka University Hospital and 83.3% (five of six patients) at the St. Marianna University Hospital; the overall percentage was 69.2% (Table 3). Among these, five patients (38.5%) showed improvement of OMDS by ≥2. One patient could not walk 10 m; therefore, the 10 m walking time was evaluated in 12 patients. The percentage of patients whose 10 m walking time improved by 10% after about 2 weeks from the start of pulse therapy was 100% (6 of 6) at the Fukuoka University Hospital and 83.3% (5 of 6) at the St. Marianna University Hospital; the overall percentage was 91.7% (11 of 12) (Table 3). Among these, three patients (25.0%) showed improvement in 10 m walking time by 30%. None of the patients showed deterioration in OMDS or 10 m walking time at the time of the above evaluation.

**TABLE 3:** Effects of the steroid therapy on lower limb motor function.

	**Changes in OMDS in response to steroid therapy**				
**Hospital name**	**≥2-grade improvement**	**1-grade improvement**	**No change**	**Deterioration**	**Total**
FUH	3 (42.9%)	1 (14.3%)	3 (42.9%)	0 (0.0%)	7
SMUH	2 (33.3%)	3 (50.0%)	1 (16.7%)	0 (0.0%)	6
Total	5 (38.5%)	4 (30.8%)	4 (30.8%)	0 (0.0%)	13

	**Changes in 10 m WT in response to steroid therapy**				
**Hospital name**	**≥30% improvement**	**30%>≥10% improvement**	**changes within 10%**	**≥10% deterioration**	**Total**

FUH	1 (16.7%)	5 (83.3%)	0 (0.0%)	0 (0.0%)	6^∗^
SMUH	2 (33.3%)	3 (50.0%)	1 (16.7%)	0 (0.0%)	6
Total	3 (25.0%)	8 (66.7%)	1 (8.3%)	0 (0.0%)	12

*^∗^One non-ambulatory patient was excluded. OMDS, osame motor disability score; FUH, Fukuoka University Hospital; SMUH, St. Marianna University Hospital; 10 m WT, 10-meter walking time.*

### Markers of Response to Steroid Therapy

**FIGURE 1:**
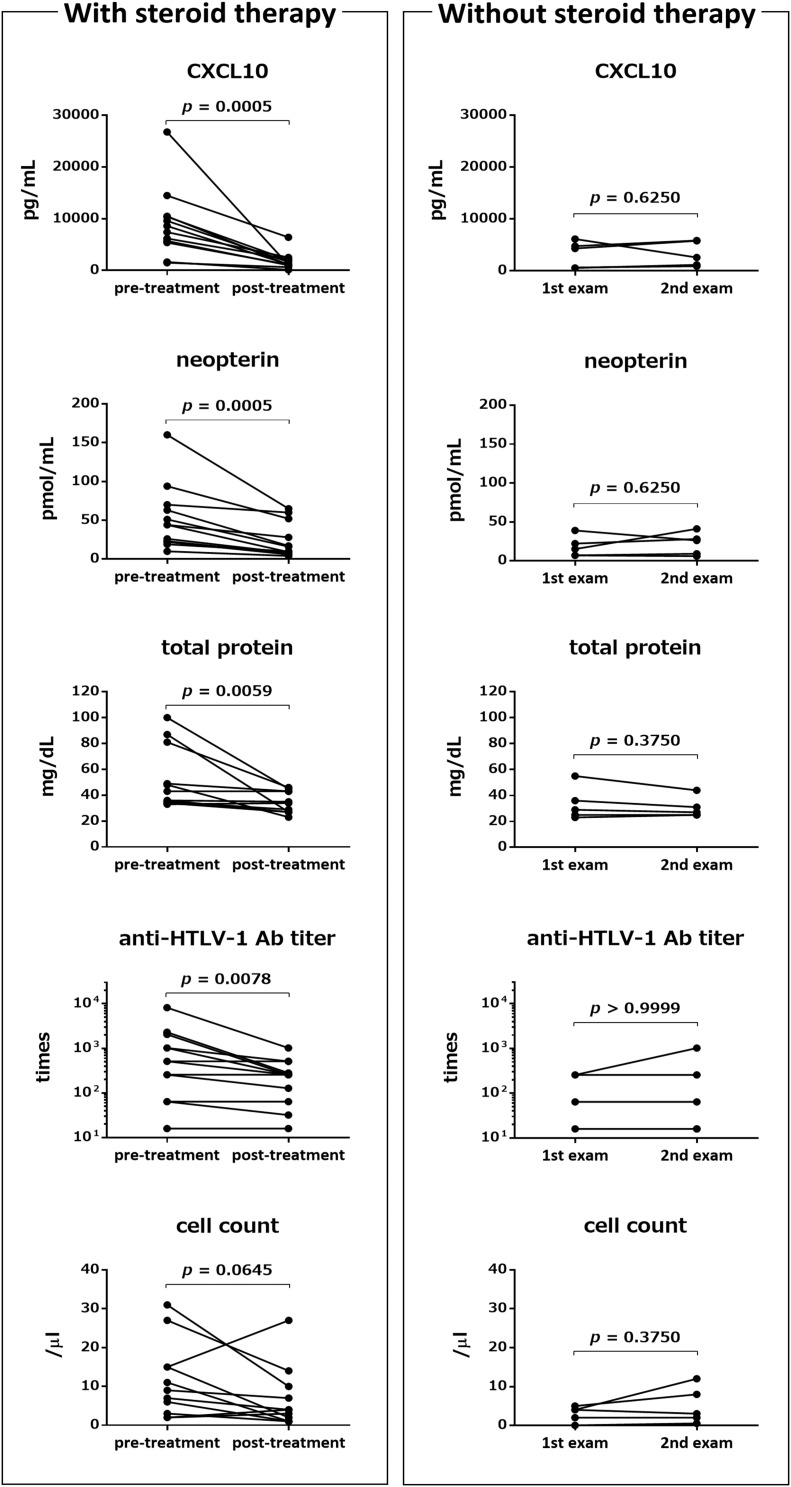
Effects of steroid therapy on Cerebrospinal fluid (CSF) markers. Left: Comparison of pre-treatment levels of the following five CSF markers with those approximately 2 weeks after steroid therapy (mean ± standard deviation (SD): 2.5 ± 0.9 weeks from the first day of pulse therapy): C-X-C motif chemokine 10 (CXCL10), neopterin, total protein, anti-HTLV-1 antibody (Ab) titer, and cell count. Post-treatment CSF markers were not available for one or two patients among the 13 patients who received methylprednisolone pulse therapy (*n* = 12: CXCL10, neopterin, and anti-HTLV-1 antibody titer; *n* = 11: total protein and cell count). Right: Comparison of the same five CSF markers between two time points (mean ± SD: 16.4 ± 5.7 months) in five patients who did not receive any steroid treatment and interferon alpha treatment. Statistical analysis was performed using a Wilcoxon signed rank test. Ab, antibody.

We compared the pre- and post-treatment levels of five CSF markers that are believed to reflect the level of spinal cord inflammation in HAM/TSP patients (*n* = 11 or 12). As shown in Figure 1 (left), the levels of CXCL10, neopterin, total protein, and anti-HTLV-1 antibody in CSF of HAM/TSP patients who received steroid therapy were significantly reduced 2 weeks after treatment, compared with the pre-treatment levels (*p* = 0.0005, *p* = 0.0005, *p* = 0.0059, and *p* = 0.0078, respectively). CSF cell counts also tended to decrease; however, the difference was not significant (*p* = 0.0645). When comparing the pre- and post-treatment levels for each hospital, significant reduction was observed only for two out of the five CSF markers (CXCL10 and neopterin) (data not shown). In contrast, none of the five markers showed a significant reduction in HAM/TSP patients (*n* = 5) who were not treated with steroids or interferon-α (Figure 1, right).

### Predictors of Response to Steroid Therapy

**FIGURE 2:**
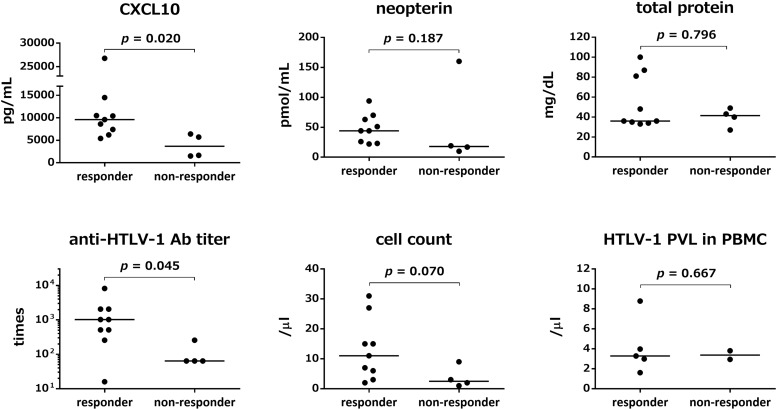
Comparison of pre-treatment marker values between responders and non-responders to steroid therapy. Responders (*n* = 9) refer to patients who showed improved Osame motor disability scores (OMDS) by one or more grade 1 month after the start of steroid therapy. Non-responders (*n* = 4) refer to patients who did not show any change in OMDS. The pre-treatment values of five CSF markers (CXCL10, neopterin, total protein, anti-HTLV-1 antibody titer, and cell count) and HTLV-1 proviral load in Peripheral blood mononuclear cells (PBMCs) were compared between responders and non-responders. Data were analyzed by Mann–Whitney U test. Ab, antibody; PVL, proviral load; PBMC, peripheral blood mononuclear cells.

HTLV-1-associated myelopathy/tropical spastic paraparesis patients who showed improvement in OMDS were defined as responders (9 out of 13 patients). In order to identify predictors of therapeutic response, we compared the pre-treatment marker levels between responders and non-responders (Figure 2). In addition to the five CSF markers described above, we also assessed the HTLV-1 proviral load in PBMCs. Pre-treatment CSF levels of CXCL10 and anti-HTLV-1 antibody titer in responders were significantly higher than those in non-responders (*p* = 0.020 and *p* = 0.045, respectively). CSF neopterin concentration was not significantly different (*p* = 0.187); however, after exclusion of one non-responder (outlier), CSF neopterin was significantly higher in responders (*p* = 0.009). CSF cell count showed a higher tendency in responders (*p* = 0.070). There was no significant difference between the two groups with respect to total protein level (*p* = 0.796); nevertheless, the total protein level was high in some of the responders. Although the number of patients who we investigated was limited, the HTLV-1 proviral load in PBMCs did not differ significantly between responders and non-responders (*p* = 0.667).

### Relationship Between Clinical Course and CSF Marker Levels

**FIGURE 3:**
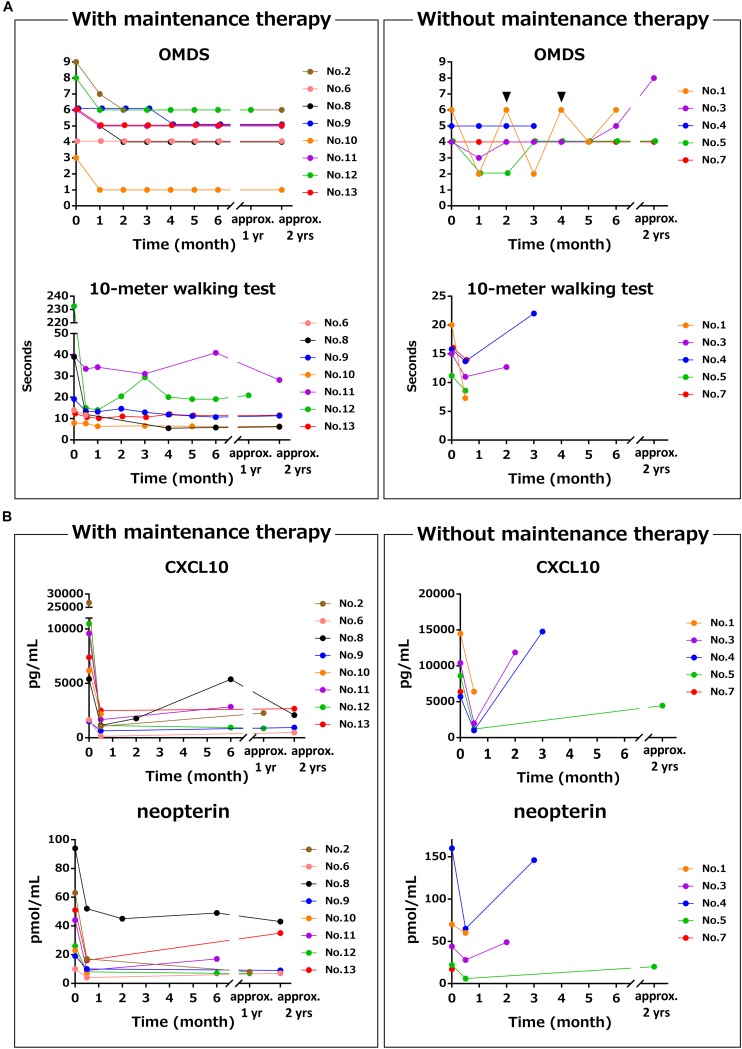
Differences in time course of motor function and CSF markers with and without maintenance therapy using oral prednisolone after methylprednisolone pulse therapy. These graphs demonstrate the time course of (A) OMDS and 10 m walking time and (B) CSF CXCL10 concentration and CSF neopterin concentration in eight HAM/TSP patients who received maintenance therapy (left) and five HAM/TSP patients who did not receive maintenance therapy (right). The data of the 10 m timed walk were obtained from seven patients, as one of eight patients who received maintenance therapy was unable to walk 10 m. Among the five patients who did not receive maintenance therapy, patient no. 1 repeatedly received methylprednisolone pulse therapy every 2 months. The arrowhead indicates the time of administration. Patient no. 4 received oral prednisolone therapy after the third CSF test because of the clinical deterioration with high levels of CXCL10 and neopterin. OMDS, Osame motor disability score.

**FIGURE 4:**
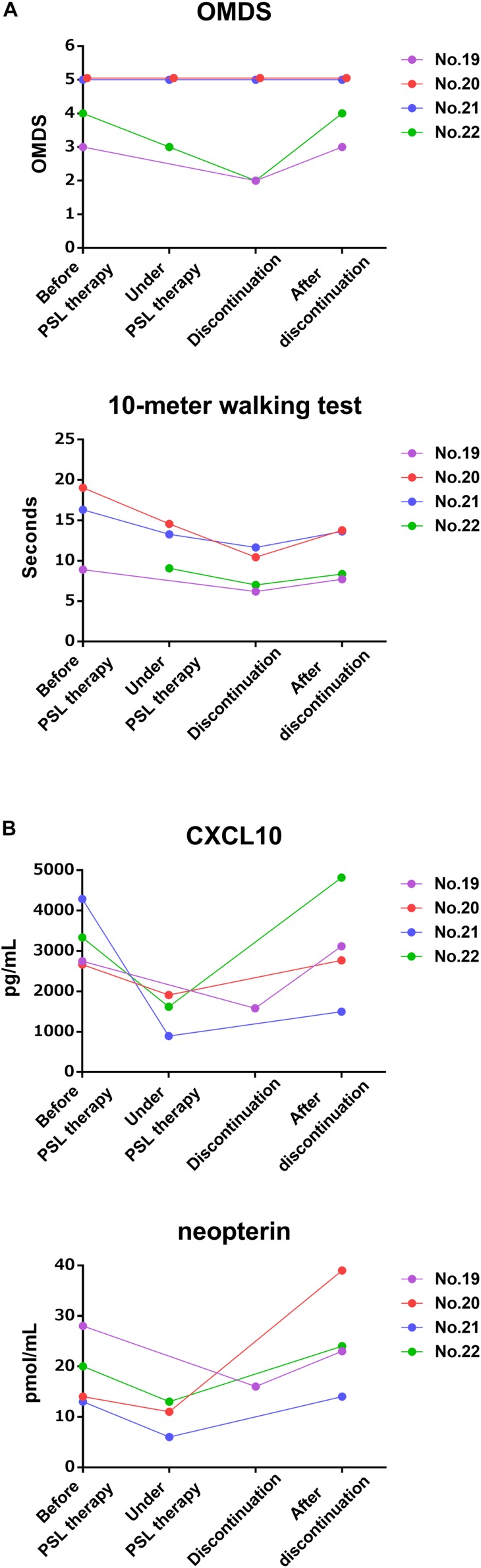
Time course of motor function and CSF markers in patients received and subsequently discontinued oral prednisolone therapy. These graphs demonstrate the time course of (A) OMDS and 10 m walking time and (B) CSF CXCL10 concentration and CSF neopterin concentration in four HAM/TSP patients treated with low-dose prednisolone from before treatment to after treatment discontinuation. The pre-treatment data of the 10 m walking time in patient no. 22 could not be obtained. OMDS, Osame motor disability score; PSL, prednisolone.

Next, the clinical course and the time course of CSF marker levels for approximately 2 years after initiation of treatment were compared between the group that received maintenance therapy with oral prednisolone after methylprednisolone pulse therapy (*n* = 8) and the group that did not receive oral prednisolone (*n* = 5). The OMDS in seven of eight HAM/TSP patients who received maintenance therapy improved compared with that at baseline; the improvement was maintained afterward in all but one patient (no. 6) who showed no change throughout 2 years (Figure 3A, upper left). Six of seven patients who were able to walk 10 m with or without walking aids showed sustained improvement in the 10 m walking time (Figure 3A, lower left). In contrast, three out of five patients who had not received maintenance therapy showed improvement in OMDS 1 month after pulse therapy; however, the OMDS subsequently deteriorated and returned to the original level in 2 or 3 months (Figure 3A, upper right). Data pertaining to 10 m walking time from 2 weeks after treatment were only available for two patients. The results showed initial improvement followed by deterioration in the 10 m walking time (Figure 3A, lower right). As shown in Table 4, there was a significant association between maintenance therapy with oral prednisolone and change in OMDS during the observation period (*p* = 0.035). We were able to obtain longitudinal data pertaining to CXCL10 and neopterin levels in CSF from seven of eight patients who received maintenance therapy and three of five patients who did not receive maintenance therapy. As shown in Figure 3B, both marker levels decreased after the pulse therapy; subsequently, there was a rapid increase in the levels in patients who did not receive maintenance therapy (Figure 3B, right) compared with those who received maintenance therapy (Figure 3B, left). The rapid increase in marker levels and the deterioration of OMDS and 10 m walking time occurred in the same time frame. Furthermore, we investigated the clinical course and the time course of CSF marker levels in four HAM/TSP patients who received and subsequently discontinued low-dose oral prednisolone therapy (Figure 4). The OMDS and 10 m walking time showed the best values at the point of treatment discontinuation and gradually deteriorated after discontinuation (Figure 4A). The CXCL10 and neopterin levels in all four patients decreased with the treatment and increased after discontinuation (Figure 4B).

**TABLE 4:** Association between maintenance therapy and change in OMDS during the observation period.

	**Change in OMDS during the observation period**		
	**Deterioration**	**No**	**Total**
		**deterioration**	
Patients with maintenance therapy	0	8	8
Patients without maintenance therapy	3	2	5

**p* = 0.035 by Fisher’s exact test.*

## Discussion

The present study demonstrated that CSF CXCL10 is a marker of therapeutic response in HAM/TSP patients. Steroid-induced reduction in CSF CXCL10 occurred simultaneously with the amelioration of clinical symptoms (Figures 1, 3, left and Table 3); conversely, withdrawal of steroid treatment increased CSF CXCL10 alongside the deterioration in clinical symptoms (Figures 3, right, 4). In addition, maintenance of reduced CSF CXCL10 by oral prednisolone therapy after pulse therapy was associated with maintenance of motor function improvement for 2 years (Figure 3 and Table 4). These data suggested that CSF CXCL10 qualifies the following criteria for a surrogate marker: “the effect of treatment on the surrogate marker correlates with its effect on the true endpoint.” In other words, treatment-induced decrease in CSF CXCL10 concentration was associated with improvement in functional prognosis. Unfortunately, the relatively short observation period (2 years) in this study is a limitation. However, our previous retrospective study has already revealed that patients with high disease activity have high CSF CXCL10 levels and poor functional prognosis after decades; conversely, patients with low disease activity have low CSF CXCL10 levels and good functional long-term prognosis (Sato et al., 2018b). Furthermore, in HAM/TSP patients who received anti-CCR4 antibody therapy, low CSF CXCL10 levels were maintained for about 10 months; during this period, clinical improvement was maintained (Sato et al., 2018a). Therefore, treatment-induced decrease in CSF CXCL10 level is likely to improve the long-term functional prognosis of HAM/TSP patients. CSF CXCL10 has been shown to be involved in the pathogenesis of HAM/TSP (Ando et al., 2013); in addition, it demonstrated a correlation with the degree of progression and long-term functional prognosis (Sato et al., 2013, 2018b). Thus, it is likely that CSF CXCL10 is a biomarker that meets all the conditions as a surrogate marker for treatment of HAM/TSP. If correct, maintenance of CSF CXCL10 at low levels should be an important therapeutic goal in order to improve long-term functional prognosis.

In addition to CSF CXCL10, CSF neopterin is a promising candidate surrogate marker. Among the CSF markers examined in this study, CSF levels of neopterin, total protein, and anti-HTLV-1 antibody titer as well as CXCL10 were also significantly reduced by steroid therapy (Figure 1). However, among these, only CSF CXCL10 and CSF neopterin were significantly reduced by steroid therapy at both university hospitals (data not shown). Steroid therapy has also been shown to reduce CSF neopterin level (Nakagawa et al., 1996; Nagai et al., 2013). Furthermore, in the present study, the chronological changes in CSF neopterin concentration with steroid treatment were similar to those in CSF CXCL10, both of which were also associated with the clinical course (Figures 3, 4). Therefore, CSF neopterin is also a potential surrogate marker for the treatment of HAM/TSP. However, since the association between CSF neopterin and HAM/TSP pathogenesis is still unclear, CSF CXCL10 is currently considered to be the most suitable surrogate marker.

There is no clear consensus on the effectiveness of methylprednisolone pulse therapy in patients with HAM/TSP. In the current study, although methylprednisolone pulse therapy improved clinical symptoms in many patients with disease activity, its effectiveness was transient in patients without steroid maintenance therapy. In addition, this study suggested that maintenance therapy with oral prednisolone is necessary to sustain this improved condition (Figure 3). Indeed, about 90% of patients who received methylprednisolone pulse therapy improved 10 m walking time by 10% or more in about 2 weeks after treatment, and about 70% of them showed improved OMDS 1 month after treatment (Table 3). However, absence of maintenance therapy led to the worsening of motor ability of their lower limbs along with an increase in CSF marker levels; the CSF marker levels returned to their baseline level within a few months. On the other hand, patients who received maintenance therapy showed persistently low CSF marker levels and sustained improvement of lower limb motor ability (Figure 3). There was a significant association between administration of maintenance therapy and the change in OMDS during the observation period (Table 4). Additionally, continuous use of low-dose prednisolone was earlier shown to improve long-term motor functional prognosis (Coler-Reilly et al., 2017). These findings suggest the benefits of low-dose oral prednisolone maintenance therapy after pulse therapy. However, the optimal duration of maintenance therapy and its safety aspects are not clear. Future prospective studies should evaluate the efficacy and safety of low-dose oral prednisolone maintenance therapy after adjusting for patient characteristics.

The present study found that CSF CXCL10 concentration and CSF anti-HTLV-1 antibody titer may help predict the response to steroid therapy. It is likely that patients with high levels of these markers represent a subgroup of patients in whom steroid therapy is likely to be particularly effective. Indeed, responders who showed a high degree of clinical improvement by steroid therapy showed significantly higher levels of CSF CXCL10 concentration and CSF anti-HTLV-1 antibody titer before the start of treatment compared with non-responders (Figure 2). As for CSF neopterin, there was no significant difference between responders and non-responders, prior to exclusion of outlier data. However, CSF neopterin is also likely to be one of the predictors of therapeutic response, as suggested by a previous study (Nagai et al., 2013). The above results suggest that steroid therapy is particularly useful for patients who exhibit high concentrations of CXCL10 and neopterin in CSF; in contrast, steroid treatment offers limited benefit for patients with low or normal levels of these CSF markers. We have already shown that HAM/TSP patients can be classified into three groups according to disease activity, which is assessed on the basis of CSF concentrations of CXCL10 and neopterin (Sato et al., 2018b) and clinical progression rate. In this context, our study underlines the importance of so-called stratified medicine, wherein the treatment strategy is decided according to the pre-treatment disease activity.

In some studies, methylprednisolone pulse therapy was not found to be very effective in HAM/TSP patients. This may be attributable to the inclusion of non-responders with low levels of inflammation in the spinal cord. Indeed, these studies did not involve assessment of pre-treatment disease activity (inflammatory markers) in the spinal cord (Duncan and Rudge, 1990; Araujo et al., 1993). Therefore, it is critical to take into account the extent of disease activity in order to predict therapeutic efficacy.

This study has mainly two limitations. The observation period of 2 years was relatively short considering the prolonged clinical course of HAM/TSP. Second, there were many missing data due to retrospective data collection. Future prospective study involving data collection over a longer period of time may provide more definitive evidence. However, a prospective study may pose a challenge as denial of maintenance therapy to patients with high disease activity is ethically difficult after the importance of maintenance therapy becomes apparent.

## Conclusion

In conclusion, the present study suggests that CSF CXCL10 may serve both as a marker and predictor of therapeutic response in patients with HAM/TSP. In addition, since it is likely that the therapeutic effect on CSF CXCL10 is associated with the effect on long-term functional prognosis of HAM/TSP patients, CSF CXCL10 has a potential to serve as a surrogate marker for treatment of HAM/TSP.

## Data Availability

The datasets generated for this study are available on request to the corresponding author.

## Ethics Statement

The studies involving human participants were reviewed and approved by the Institutional Review Board of St. Marianna University School of Medicine (#1646) and Fukuoka University Hospital (#14-2-08). The patients/participants provided their written informed consent to participate in this study.

## Author Contributions

KT, YY, and YT conceived and designed the study. KT, TS, JT, SF, JY, and YY collected the clinical data. KT and TS analyzed the results. KT, TS, NY, NA, JY, AC-R, MN, YH, and YY drafted and corrected the manuscript. All authors read and approved the final manuscript.

## Conflict of Interest Statement

The authors declare that the research was conducted in the absence of any commercial or financial relationships that could be construed as a potential conflict of interest.
